# Association Between Epicardial Adipose Tissue Thickness and Early Arrhythmia Recurrence After Electrical Cardioversion for Atrial Fibrillation and Atrial Flutter in Patients With and Without Heart Failure—An Observational Study

**DOI:** 10.3390/medicina62061130

**Published:** 2026-06-10

**Authors:** Fabio Kadum, Koraljka Benko, Lara Batičić, Ana Petretić

**Affiliations:** 1 Clinic for Cardiovascular Diseases, University Hospital Center Rijeka, 51000 Rijeka, Croatia; bkoraljka@yahoo.com (K.B.); aantonic326@gmail.com (A.P.); 2 Faculty of Medicine, University of Rijeka, 51000 Rijeka, Croatia; lara.baticic@uniri.hr

**Keywords:** atrial fibrillation, atrial flutter, electrical cardioversion, epicardial adipose tissue, echocardiography, heart failure

## Abstract

*Background and Objectives*: Atrial fibrillation (AF) and atrial flutter are associated with significant morbidity. While electrical cardioversion (ECV) is a standard treatment for sinus rhythm restoration, early arrhythmia recurrence is common. Epicardial adipose tissue (EAT) is a metabolically active depot linked to atrial remodeling, yet its role in predicting post-ECV outcomes remains unclear. This study aimed to evaluate the association between EAT thickness measured by echocardiography and early arrhythmia recurrence following elective ECV in patients with and without heart failure (HF). *Materials and Methods*: A single-center observational study was conducted including 76 patients (38 with previously diagnosed HF) who underwent successful elective ECV (eECV) for AF or atrial flutter. Preprocedural transesophageal echocardiography was performed to exclude thrombi, while EAT thickness was assessed via transthoracic echocardiography (TTE). The primary outcome was early arrhythmia recurrence (within 30 days), and a secondary outcome was arrythmia recurrence in the HF subgroup. Data were analyzed using univariable and multivariable logistic regression, receiver operating characteristic (ROC) analysis, and subgroup analysis in patients with HF. *Results*: Arrhythmia recurrence occurred in 19 patients (25.0%) within the follow-up period. Mean EAT thickness did not differ significantly between patients with and without recurrence (3.74 ± 1.41 mm vs. 3.58 ± 1.78 mm; *p* = 0.42). EAT thickness did not emerge as a significant predictor of recurrence in univariable or multivariable models, demonstrating poor discriminative ability (AUC = 0.56). Similar findings were observed in the HF subgroup (OR = 0.94, *p* = 0.75; AUC = 0.49). Conversely, left atrial (LA) size was significantly associated with recurrence (OR = 2.76, *p* = 0.043). While EAT thickness correlated with body mass index and HF, it did not predict immediate rhythm outcomes. *Conclusions*: In our study, measurement of EAT thickness via TTE was not associated with early arrhythmia recurrence after eECV, including in patients with concomitant HF. These findings suggest that early post-cardioversion stability may depend more on established structural atrial remodeling, especially by LA enlargement, than on linear EAT thickness. Further research utilizing advanced volumetric imaging is warranted to clarify the role of epicardial adiposity in atrial arrhythmogenesis.

## 1. Introduction

Atrial fibrillation (AF) and atrial flutter are the most prevalent sustained cardiac arrhythmias encountered in clinical practice, representing a major public health challenge associated with substantial morbidity and mortality [1,2]. According to the 2024 ESC Guidelines, the clinical focus has shifted towards an integrated “AF-CARE” framework (C—control of risk factors and comorbidities; A—avoidance of stroke and thromboembolism; R—reduction in symptoms through rhythm and rate control; E—evaluation and dynamic reassessment) [1]. In addition to an increased risk of thromboembolic events, these arrhythmias are associated with impaired quality of life, worsening heart failure (HF), and increased healthcare burden [1,3].

In symptomatic patients, elective electrical cardioversion (eECV) remains a fundamental intervention for the restoration of sinus rhythm [4]. However, despite high initial success rates, early arrhythmia recurrence remains a common clinical challenge, necessitating the identification of reliable predictors for rhythm stability [3,4].

The mechanisms underlying recurrence are complex and rooted in atrial remodeling—a process involving structural, electrical, and metabolic alterations [5]. While left atrial (LA) enlargement and fibrosis have long been recognized as central contributors, epicardial adipose tissue (EAT) has emerged as a significant player in the development of the arrhythmogenic substrate [5,6]. EAT is a metabolically active visceral fat depot that secretes multiple proinflammatory cytokines (e.g., IL-6, TNF-α) and profibrotic mediators that contribute to oxidative stress and myocardial fibrosis [6,7].

While computed tomography and magnetic resonance imaging enable precise volumetric quantification, transthoracic echocardiography (TTE) remains a non-invasive, cost-effective, and routinely applicable method in everyday clinical practice [8]. Echocardiographic assessment of EAT thickness represents a practical surrogate for evaluating epicardial adiposity [8,9]. Previous studies have demonstrated associations between increased EAT and arrhythmia recurrence following catheter ablation [10,11]. Although EAT thickness has been investigated as a predictor of ECV success [12], data focusing specifically on early post-ECV recurrence are scarce, particularly in patients with concomitant heart failure (HF), where EAT may serve as a distinct phenotypic marker [13]. Therefore, the aim of the present study was to evaluate the association between echocardiographically measured EAT thickness and early arrhythmia recurrence after elective ECV, with a specific focus on the HF subgroup.

## 2. Materials and Methods

This single-center observational study was conducted at the Clinic for Cardiovascular Diseases, Clinical Hospital Center Rijeka. A total of 82 patients with atrial fibrillation (AF) or atrial flutter who were scheduled for elective electrical cardioversion (eECV) between September 2025 and February 2026 were included in the study. All patients were adequately anticoagulated and underwent preprocedural transesophageal echocardiography (TEE) to exclude left atrial appendage thrombus. Patients without identified thrombi subsequently underwent eECV and were included in the final analysis. Patients with unsuccessful cardioversion or those lost to follow-up were excluded. Regarding the eECV, all patients underwent the procedure under propofol sedation using an anterolateral paddle position. Biphasic synchronized shocks were delivered according to standard institutional practice. Recorded procedural variables included propofol dose, number of shocks required for successful cardioversion, cumulative energy used and use of antiarrhythmic drugs prior to cardioversion.

The primary objective was to evaluate the association between epicardial adipose tissue (EAT) thickness and early arrhythmia recurrence—within 30 days, after successful eECV. A secondary objective was to assess this relationship specifically in a subgroup of patients with heart failure (HF), including those with reduced (HFrEF), mildly reduced (HFmrEF), preserved (HFpEF), and improved (HFimpEF) ejection fraction.

Clinical and echocardiographic data were obtained on the day of the scheduled eECV. Clinical characteristics included sex (male/female), age (years), body mass index (BMI, kg/m^2^), and arterial hypertension (yes/no). Arrhythmia type was classified as paroxysmal AF, persistent AF, long-standing persistent AF, paroxysmal atrial flutter, persistent atrial flutter, or long-standing persistent atrial flutter. Symptoms were categorized as asymptomatic, palpitations, dyspnea, or chest pain. Left atrial (LA) size (parasternal short-axis view) was categorized as <40 mm, 40–49 mm, 50–59 mm, or ≥60 mm. EAT thickness was measured via transthoracic echocardiography (TTE), with the patient in left lateral decubitus position, from the parasternal long-axis view, perpendicular to the right ventricular free wall at the end of systole. It was recorded as a continuous variable (mm). For exploratory purposes, EAT thickness was also categorized into three groups: ≤3 mm, 4–5 mm, and ≥6 mm.

The method used to detect arrythmia recurrence was a routine ECG for all the patients on the follow up visit 30 days after eECV.

### Statistical Analysis

Continuous variables are expressed as mean ± standard deviation (SD), and categorical variables as absolute numbers and percentages. Due to the sample size and potential non-normal distribution, the Mann–Whitney U test was used for continuous variables, while the chi-square or Fisher’s exact test was used for categorical data.

Univariable and multivariable logistic regression analyses were performed to identify predictors of recurrence, with results reported as odds ratios (ORs) with 95% confidence intervals (CIs). The multivariable model included BMI, LA size, and HF status as covariates. Receiver operating characteristic (ROC) analysis was used to assess the discriminative ability (AUC) of EAT thickness. Correlations were assessed using Spearman’s rank correlation (rho). Statistical significance was set at *p* < 0.05.

## 3. Results

A total of 76 patients with available one-month rhythm follow-up data were included in the final analysis. The mean age of the study population was 68.5 ± 8.3 years; 55.6% of patients were male and 43.4% were female. Mean body mass index (BMI) was 29.8 ± 5.6 kg/m^2^. The most common arrythmia type was persistent atrial fibrillation (AF) present in 86.8% of patients. Regarding symptoms, dyspnea, present either alone or in combination with palpitations, was most frequently reported (59.2% of patients). A total of 25% of patients were asymptomatic. Arrhythmia recurrence within 30 days after elective electrical cardioversion (eECV) was documented in 19 patients (25.0%), while 57 patients (75.0%) maintained sinus rhythm during the follow-up period.

### 3.1. Epicardial Adipose Tissue Thickness and Arrhythmia Recurrence

Patients without arrhythmia recurrence had a mean EAT thickness of 3.58 ± 1.78 mm, while patients with recurrence had a mean EAT thickness of 3.74 ± 1.41 mm. No statistically significant difference in EAT thickness was observed between the groups (Mann–Whitney U test, *p* = 0.42) (Table 1).

To evaluate a potential threshold effect, patients were categorized into three predefined EAT groups. Among patients with EAT ≤ 3 mm, arrhythmia recurrence occurred in 9 patients (21.4%). In the 4–5 mm group, recurrence occurred in 8 patients (33.3%), while 2 patients (20.0%) with EAT ≥ 6 mm experienced recurrence. No statistically significant difference in recurrence rates was observed between categories (chi-square test, *p* = 0.52) (Table 2).

### 3.2. Logistic Regression and ROC Analysis

In univariable logistic regression analysis, EAT thickness as a continuous variable was not associated with arrhythmia recurrence (OR per 1 mm increase 1.06; 95% CI 0.78–1.43; *p* = 0.72). Following adjustment for BMI, left atrial (LA) size, and heart failure (HF) status in the multivariable model, EAT thickness remained non-significant (OR 0.99; 95% CI 0.68–1.44; *p* = 0.97). In contrast, LA size was significantly associated with recurrence (OR 2.76; 95% CI 1.03–7.40; *p* = 0.043) (Table 3).

Receiver operating characteristic (ROC) analysis demonstrated limited discriminative ability of EAT thickness for prediction of arrhythmia recurrence, with an area under the curve (AUC) of 0.56, indicating poor predictive performance.

### 3.3. Procedural Variables and Arrythmia Recurrence

Exploratory analyses demonstrated no significant association between procedural variables and early arrhythmia recurrence. Mean propofol dose did not differ significantly between patients with and without recurrence (65.8 mg vs. 68.3 mg, *p* = 0.79). Similarly, no significant differences were observed regarding the number of shocks required for successful cardioversion (1.00 vs. 1.07, *p* = 0.32). Also, cumulative delivered energy, calculated as maximal delivered energy multiplied by the number of attempts, was not associated with recurrence (200.0 J vs. 201.5 J, *p* = 0.23). Exploratory analysis of antiarrhythmic premedication also demonstrated no association with recurrence. Recurrence occurred in 24.2% of patients without antiarrhythmic premedication and in 25.0% of patients receiving antiarrhythmic pretreatment (*p* = 1.00). The antiarrhythmic drugs included amiodarone, flecainide and propafenone.

### 3.4. Heart Failure Subgroup Analysis

In the HF subgroup (n = 38), arrhythmia recurrence occurred in 12 patients (31.6%). Mean EAT thickness did not differ significantly between patients with and without recurrence (3.92 ± 1.38 mm vs. 4.12 ± 1.99 mm; *p* = 0.95) (Table 4). Logistic regression within this subgroup showed no association (OR 0.94; 95% CI 0.63–1.39; *p* = 0.75), and ROC analysis confirmed a lack of predictive value (AUC = 0.49). Given the limited sample size and low number of events, these analyses should be considered exploratory and hypothesis-generating. Stratified analyses according to HF phenotype were not feasible due to insufficient statistical power.

### 3.5. Correlation Between EAT Thickness and Markers of Remodeling

Correlation analyses (Table 5) revealed a moderate positive correlation between EAT thickness and BMI (rho = 0.40; *p* = 0.0003) and a significant association with the presence of HF (rho = 0.27; *p* = 0.019). A weak positive trend toward larger LA size with increasing EAT thickness was observed (rho = 0.19; *p* = 0.099), while no significant associations were found for age or hypertension.

## 4. Discussion

Electrical cardioversion (ECV) is a widely used and well-established procedure for restoring normal sinus rhythm in patients with atrial fibrillation (AF) and atrial flutter. During the procedure, a synchronized electrical shock is delivered to the heart under short-term anesthesia or deep sedation, allowing the atria to resume coordinated electrical activity. ECV is one of the most effective rhythm-control strategies available in routine clinical practice. Previous studies have shown that sinus rhythm can be restored successfully in approximately 90% of patients, which makes ECV considerably more effective than pharmacological cardioversion, where antiarrhythmic drugs achieve success in only 50–70% of cases [14].

Although the immediate success rate of ECV is high, maintaining sinus rhythm over time remains a major clinical challenge. Many patients experience recurrence of AF or atrial flutter shortly after the procedure. Earlier studies reported that approximately 40% of patients develop recurrent atrial arrhythmias within the first three months following successful cardioversion [15]. This high recurrence rate reflects the complex and often persistent pathophysiological abnormalities underlying atrial arrhythmias, including electrical remodeling, structural changes, inflammation, and fibrosis. These observations highlight the importance of identifying reliable predictors of recurrence in order to improve patient selection, tailor treatment strategies, and optimize long-term clinical outcomes.

The principal finding of the present study was that epicardial adipose tissue (EAT) thickness, measured by transthoracic echocardiography (TTE), was not significantly associated with early recurrence of AF or atrial flutter after elective ECV. Statistical analysis showed no meaningful relationship between EAT thickness and arrhythmia recurrence within 30 days (*p* = 0.42), and the discriminatory performance was poor, with an area under the receiver operating characteristic curve (AUC) of 0.56, which is only slightly better than chance. Importantly, this lack of association remained consistent when EAT was analyzed as a continuous variable, when patients were categorized according to predefined cutoff values, and when the analysis was restricted to the subgroup of patients with heart failure (HF).

The relationship between EAT and atrial arrhythmias has attracted increasing scientific interest because EAT is now recognized as a metabolically active visceral fat depot rather than a passive storage compartment. Located between the myocardium and the visceral pericardium, EAT shares the same microcirculation as the underlying heart muscle and is not separated from it by a fascial barrier. As a result, EAT can directly affect the myocardium through paracrine and vasocrine signaling. It secretes proinflammatory cytokines, such as interleukin-6 (IL-6) and tumor necrosis factor alpha (TNF-α), as well as adipokines and profibrotic mediators that may promote inflammation, oxidative stress, fibrosis, and electrical remodeling [6]. These mechanisms are believed to contribute to the long-term development of an arrhythmogenic substrate. However, our findings suggest that very early recurrence after cardioversion may depend more strongly on short-term factors such as transient atrial stunning, residual electrical instability, and pre-existing structural remodeling than on the chronic inflammatory influences potentially mediated by EAT.

An important methodological aspect of this study was the use of TTE to assess EAT thickness. In accordance with established protocols, EAT was measured on the free wall of the right ventricle in the parasternal long-axis view at end-systole, when epicardial fat is most clearly visualized [8]. TTE offers several practical advantages, including wide availability, low cost, and the absence of ionizing radiation. Moreover, echocardiographic measurements correlate reasonably well with epicardial fat volumes obtained by computed tomography (CT) and magnetic resonance imaging (MRI). Nevertheless, echocardiography provides only a linear two-dimensional estimate of EAT thickness and does not capture the full three-dimensional distribution of epicardial fat. This methodological limitation may partly explain the discrepancy between our findings and previous studies demonstrating an association between EAT and arrhythmia recurrence after pulmonary vein isolation (PVI), in which EAT was quantified using advanced imaging techniques capable of measuring total and periatrial EAT volume [10,11]. These modalities likely provide a more accurate representation of the local inflammatory burden surrounding the left atrium (LA) and pulmonary veins, which are critical anatomical regions for the initiation and maintenance of AF. Therefore, the absence of a significant association in our study may reflect limitations of EAT quantification by TTE rather than a true lack of pathophysiological relevance of epicardial adiposity in AF recurrence. A recent comprehensive review of multimodality cardiovascular imaging highlighted the complementary roles of echocardiography, CT and cardiac MRI in the evaluation of EAT, emphasizing that different imaging modalities provide distinct information regarding EAT distribution, volume and cardiovascular risk stratification [16].

Recognition of EAT as a potentially modifiable contributor to atrial remodeling has stimulated growing interest in therapies targeting cardiometabolic pathways. Previous studies have demonstrated that sustained weight loss and intensive management of cardiovascular risk factors can significantly reduce AF burden and improve rhythm control outcomes [17]. In addition, emerging evidence suggests that newer pharmacological agents, including glucagon-like peptide-1 (GLP-1) receptor agonists and sodium-glucose cotransporter-2 (SGLT2) inhibitors, may reduce EAT volume and attenuate its inflammatory activity [17,18]. Although our study did not identify a direct association between EAT thickness and early arrhythmia recurrence, the significant relationships observed between EAT, body mass index (BMI), and HF support the concept that epicardial adiposity remains clinically relevant as an indicator of broader cardiometabolic and structural remodeling.

In contrast to EAT thickness, LA size was significantly associated with arrhythmia recurrence after ECV (odds ratio [OR] 2.76, *p* = 0.043). This finding reinforces the well-established role of structural atrial remodeling in determining rhythm stability after cardioversion. Enlargement of the LA is widely regarded as a surrogate marker of advanced atrial myopathy, reflecting cumulative effects of pressure and volume overload, fibrosis, and chronic electrical remodeling [18]. A larger atrial chamber provides a greater surface area that can sustain multiple simultaneous re-entrant wavelets, while atrial wall stretch promotes conduction heterogeneity and interstitial fibrosis, both of which disrupt normal impulse propagation and facilitate the recurrence of AF and atrial flutter [19].

## 5. Conclusions

In conclusion, this single-center observational study demonstrated that epicardial adipose tissue (EAT) thickness, as measured by transthoracic echocardiography, was not a significant predictor of early arrhythmia recurrence following elective electrical cardioversion in patients with atrial fibrillation or atrial flutter. This lack of association remained consistent regardless of whether EAT was analyzed as a continuous or categorical variable, even within the heart failure (HF) subgroup. Conversely, left atrial size was significantly associated with arrhythmia recurrence, reinforcing the critical role of structural atrial remodeling in the maintenance of atrial arrhythmias. While EAT thickness correlated with body mass index and HF—suggesting its role as a marker of systemic cardiometabolic burden—it appears that early post-cardioversion rhythm stability depends more on established structural changes than on echocardiographic EAT measures alone. The findings suggest that linear EAT assessment via echocardiography may have limited clinical utility in predicting immediate procedural outcomes. Future research should prioritize advanced volumetric imaging and regional EAT assessment to further elucidate the complex relationship between epicardial adiposity and atrial arrhythmogenesis.

### Limitations

Several important limitations of the present analysis should be acknowledged. First, this was a single-center observational study with a relatively small sample size, particularly regarding the number of recurrence events. The present study should therefore be interpreted as exploratory and hypothesis-generating. In addition, no formal preregistered study protocol or a priori sample size calculation was performed. This leads to limited generalizability of the study. Only 19 patients experienced arrhythmia recurrence within 30 days after electrical cardioversion (ECV), which limits the statistical power and increases the possibility of a type II error; thus, weaker associations may not have been detected. Consequently, the absence of a significant association between epicardial adipose tissue (EAT) thickness and recurrence should be interpreted cautiously and regarded as inconclusive rather than definitive.

Second, epicardial adipose tissue (EAT) thickness was assessed using transthoracic echocardiography (TTE) rather than computed tomography or cardiac magnetic resonance imaging. Although TTE is clinically accessible, it represents a linear measurement and may not fully reflect total EAT volume or regional distribution. Consequently, our negative findings may partly reflect methodological limitations of TTE-based EAT assessment. Third, the follow-up period was limited to one month after ECV. EAT may be more strongly associated with chronic atrial remodeling and long-term recurrence rather than very early outcomes. Fourth, despite multivariable adjustment, residual confounding cannot be excluded due to the observational design. Finally, the heart failure (HF) subgroup analysis (n = 38) did not distinguish HF phenotype due to small sample size and should therefore be considered exploratory and hypothesis-generating rather than definitive.

## Figures and Tables

**Table 1:** EAT thickness according to arrhythmia recurrence status (EAT, epicardial adipose tissue).

Outcome	n	EAT Thickness (mm), Mean ± SD	*p*-Value
No recurrence	57	3.58 ± 1.78	0.42
Recurrence	19	3.74 ± 1.41	

**Table 2:** Arrhythmia recurrence according to EAT categories (EAT, epicardial adipose tissue).

EAT Category	No Recurrence	Recurrence	Recurrence Rate (%)
≤3 mm	33	9	21.4%
4–5 mm	16	8	33.3%
≥6 mm	8	2	20.0%

**Table 3:** Multivariable logistic regression analysis for prediction of arrhythmia recurrence (BMI, body mass index; EAT, epicardial adipose tissue; CI, confidence interval; OR, odds ratio).

Variable	OR	95% CI	*p*-Value
EAT thickness (per 1 mm)	0.99	0.68–1.44	0.97
BMI	0.98	0.88–1.10	0.77
Left atrial size	2.76	1.03–7.40	0.043
Heart failure	1.62	0.51–5.19	0.41

**Table 4:** EAT thickness and arrhythmia recurrence in patients with heart failure (EAT, epicardial adipose tissue).

Outcome	n	EAT Thickness (mm), Mean ± SD	*p*-Value
No recurrence	26	4.12 ± 1.99	0.95
Recurrence	12	3.92 ± 1.38	

**Table 5:** Correlation between EAT thickness and clinical parameters (BMI, body mass index; EAT, epicardial adipose tissue).

Parameter	Spearman rho	*p*-Value
BMI	0.40	0.0003
Left atrial size	0.19	0.099
Heart failure	0.27	0.019
Age	0.09	0.46
Hypertension	0.21	0.070

## Data Availability

Original data available upon request.

## Abbreviations

The following abbreviations are used in this manuscript:

AF	atrial fibrillation
BMI	body mass index
CI	confidence interval
EAT	epicardial adipose tissue
ECV	electrical cardioversion
eECV	elective electrical cardioversion
HF	heart failure
HFpEF	heart failure with preserved ejection fraction
HFimpEF	heart failure with improved ejection fraction
HFmrEF	heart failure with mildly reduced ejection fraction
HFrEF	heart failure with reduced ejection fraction
LA	left atrium
SD	standard deviation
TEE	transesophageal echocardiography
TTE	transthoracic echocardiography
